# Identification of DNA repair gene signature and potential molecular subtypes in hepatocellular carcinoma

**DOI:** 10.3389/fonc.2023.1180722

**Published:** 2023-05-16

**Authors:** Yi Bai, Jinyun He, Yanquan Ma, He Liang, Ming Li, Yan Wu

**Affiliations:** ^1^ Department of Critical Care Medicine, Panjin Liaoyou Baoshihua Hospital, Liaoning, China; ^2^ Department of hepatobiliary surgery, Panjin Liaoyou Baoshihua Hospital, Liaoning, China; ^3^ Department of integrated Chinese and Western medicine, Panjin Liaoyou Baoshihua Hospital, Liaoning, China; ^4^ Fuxin Municipal Discipline Inspection Commission, Liaoning, China; ^5^ Department of rheumatology and immunology, Panjin Liaoyou Baoshihua Hospital, Liaoning, China

**Keywords:** hepatocellular carcinoma, DNA repair gene, prognosis, molecular subtypes of HCC, immune infiltrition

## Abstract

DNA repair is a critical factor in tumor progression as it impacts tumor mutational burden, genome stability, PD-L1 expression, immunotherapy response, and tumor-infiltrating lymphocytes (TILs). In this study, we present a prognostic model for hepatocellular carcinoma (HCC) that utilizes genes related to the DNA damage response (DDR). Patients were stratified based on their risk score, and groups with lower risk scores demonstrated better survival rates compared to those with higher risk scores. The prognostic model’s accuracy in predicting 1-, 3-, and 5-year survival rates for HCC patients was analyzed using receiver operator curve analysis (ROC). Results showed good accuracy in predicting survival rates. Additionally, we evaluated the prognostic model’s potential as an independent factor for HCC prognosis, along with tumor stage. Furthermore, nomogram was employed to determine the overall survival year of patients with HCC based on this independent factor. Gene set enrichment analysis (GSEA) revealed that in the high-risk group, apoptosis, cell cycle, MAPK, mTOR, and WNT cascades were highly enriched. We used training and validation datasets to identify potential molecular subtypes of HCC based on the expression of DDR genes. The two subtypes differed in terms of checkpoint receptors for immunity and immune cell filtration capacity.Collectively, our study identified potential biomarkers of HCC prognosis, providing novel insights into the molecular mechanisms underlying HCC.

## 1 Introduction

According to the 2018 Global Cancer Statistics report, Hepatocellular Carcinoma (HCC) ranks 6th among malignancies and is the 4th leading cause of cancer-related mortality (1, 2). Despitesignificant advancements in HCC treatment, the outcomes are still unsatisfactory (3, 4). Therefore,identifying novel therapeutic targets and diagnostic biomarkers for HCC is crucial to improve patientprognosis. The DNA damage response (DDR) pathway is considered a potential source of therapeutictargets as damaged DNA is a hallmark of cancerous cells (5).

Research studies have reported that genes involved in DDR pathways, such as nucleotide and base excision and mismatch repair, are aberrantly expressed during cancer development and progression (6–10). Dysregulated DDR is associated with increased genome instability in HCC cells and has asignificant impact on patient prognosis (11).

The use of high-throughput sequencing technology has become increasingly prevalent in recentyears, and sequencing data and clinical follow-up information can be downloaded from many cancerdatabases. In this study, we downloaded the hepatocellular carcinoma dataset from TCGA and GEOdatabases to explore the prognostic potential of DNA damage response (DDR)-linked genes inhepatocellular carcinoma (HCC) and develop a risk model.

We identified 150 DDR-related genes from the MSigDB database and constructed an 11-gene HCC prognostic signature using univariate Cox regression and random forest analyses. The robustness ofthe model was validated through internal and external validation. Additionally, we used Gene SetEnrichment Analysis (GSEA) to identify potential pathways associated with the risk model in HCCand analyzed the correlation between clinical traits and the risk score. Finally, we identified andvalidated two molecular subtypes of HCC using DDR gene expression. Our findings provide novelinsights into the molecular mechanisms of HCC and establish an independent DDR gene-basedprognostic signature. The use of this signature could aid in personalized therapy and improve clinicaldecision-making for HCC patients. With the increasing availability of sequencing data, this studyprovides a useful example of how these data can be utilized to better understand the underlyingbiology of cancer and improve patient outcomes.

## 2 Methods

### 2.1 Data collection

We obtained clinical data and gene expression information for HCC samples from the ICGC-LIRI (https://dcc.icgc.org/) and TCGA-LIHC (https://portal.gdc.cancer.gov/) datasets. Genes linked toDNA damage response (DDR) were collected from MSigDB, V7.1 (https://www.gseamsigdb.org/gsea/msigdb), and only those genes present in both datasets were retained for furtheranalysis.

### 2.2 Risk signature construction

We utilized univariate and multivariate Cox regression analyses to identify DDR-linked genes in the

LIRI-JP and LIHC datasets. To calculate the risk score for each patient, we used the equation: (Exp i * β i), where Expi represents the expression level of prognostic genes and β i represents the coefficient of cox regression for each prognostic gene. The median score was used to classify patients into high and low-risk groups, and survival differences were calculated using the “survival” and “survminer” packages. To determine the accuracy of the risk model for 1-, 3-, and 5-year survival, we utilized the “SurvivalROC” package (https://cran.rproject.org/web/packages/survivalROC/index.html). We also employed univariate and multivariate cox regression analyses to determine the prognostic independence of clinical features and the risk score. Potential pathways linked to low and high-risk groups were identified by GSEA, using c2.cgp.v7.1.symbols.gmt as the reference gene set.

### 2.3 Nomogram and DCA curve construction

We constructed a nomogram utilizing independent prognostic factors, and analyzed the benefit of the prognostic factor using decision curve analysis. The discriminative ability of the nomogram was assessed using a calibration plot with the bootstrap approach and 1,000 replications (12). Furthermore, we evaluated the benefit of the prognostic factor using decision curve analysis (13).

### 2.4 Consensus clustering

We utilized the “ConsensusClusterPlus” R package (with 50 iterations and 80% resampling samples) to group patients into distinct clusters based on DDR-related genes, with the aim of determining molecular subtypes of HCC (14). Principal components analysis (PCA) was employed to distinguish between various LIHC subgroups, and all analyses were validated using the LIRI-JP dataset.

### 2.5 Immune infiltration analysis

We evaluated the enrich score of immune cells and infiltration levels of 28 immune cells for each sample in both high- and low-risk groups using the ssGSEA algorithm, which was implemented using the “GSVA” R package (15–17). Furthermore, we analyzed the expression of immune checkpoint genes in both groups.

### 2.6 Cell culture and transfection

Human HCC cell lines Hep G2 and MHCC-97H were purchased from (National collection of authenticated cell culture, Shanghai, CN), and incubated at 37 °C C with 5% CO2 in a humidity saturated environment. Cells were cultured in DMED (Hyclone, LA, USA) and supplied with 10% fetal bovine serum (BI, Israel), anti biotics (0.1 U/l penicillin and 100 g/l streptomycin). DGUOK siRNA were obtained from RiboBio Co., Ltd. (Guangzhou, China). The siRNA was dissolved in DEPC-treated water. Lipofectamine 2000 reagent (Invitrogen, CA, USA) were used for transfection according to the manufacturer’s protocol. The solutions were mixed together and incubated at room temperature for 30 minutes. 30 nM siRNA was added into each well andincubated at 37 °C

### 2.7 Hoechst staining

Cell apoptosis was observed by the morphological changes of the cell nucleus (chromatin agglutination or DNA fragmentation). Cells were treated with si-NC or si-DGUOK, and washed with

PBS twice, Hoechst 33258 (1 μg/ml) was added for 20 min at room temperature avoiding light.Images were gathered by fluorescence microscope (Nikon, Japan)

### 2.8 Cell viability assays

Cells were seeded in 96-well plates at 10,000 cells per well, and cultured for 24h. They were treated with si-NC or si-DGUOK. Then CCK-8 were added to each plate, absorbance was measured at 450 nm using a FLUOstar Omega microplate reader (BMG Labtech). Cell viability of samples wascalculated according to the manufacturer’s instructions

### 2.9 Statistical analyses

The statistical analysis was performed using R (https://www.r-project.org/). Kaplan-Meier (KM)method was employed to analyze the survival data and a p-value less than 0.05 was consideredstatistically significant.

## 3 Results

### 3.1 Identification of survival-related DDR risk model

150 DDR-related genes data were obtained from the TCGA dataset, which consisted of 343 HCCsamples. Using univariate Cox regression analysis, 37 prognostic genes that affect the survival ofpatients with HCC were identified. For developing a risk model, stepwise multivariate Coxregression analysis was conducted and 11 genes (AAAS, CANT1, CLP1, DGUOK, GTF2B,GTF2H1, NCBP2, POLA1, POLE4, POLR2D, and POLR2E) were selected. The risk score for eachpatient was calculated using the following method and computation: AAAS * -0.022 + CANT1 *0.016 + CLP1 * -0.098 + DGUOK * -0.016 + GTF2B*0.018 + GTF2H1 * 0.034 + NCBP2 * 0.042 +POLA1 * 0.089 + POLE4 * 0.015 + POLR2D * 0.047 + POLR2E * 0.007.

**Figure 1:**
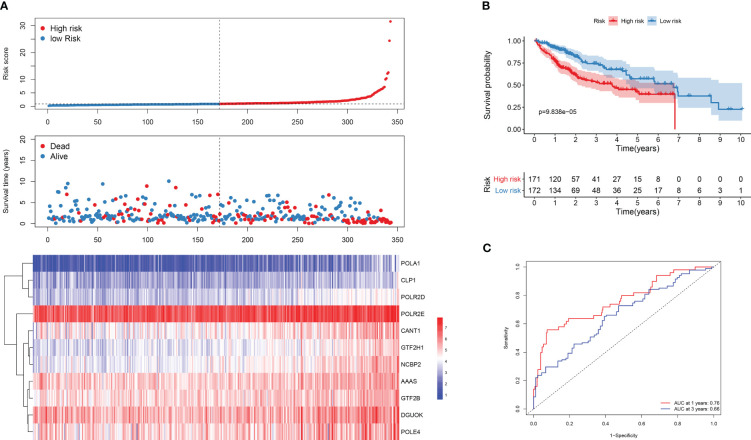
Survival analysis of DDR genes in TCGA dataset. **(A)** Risk score plot for the DDR signature. Upper panel is demonstrating the risk score distribution, middle panel is for case distribution, and the lower panel indicates the level of expression of 11 DDR genes. **(B)** KM survival curves of bothgroups. **(C)** ROC curve analysis of the risk gene signature.

The patients were then divided into low and high-risk groups based on the median risk score. Asshown in **Figure 1A**, patients in the low-risk group had a longer expected survival rate compared tothose in the high-risk group. Furthermore, KM analysis confirmed better prognosis in the low-riskgroup than in the high-risk group (p<0.001) (**Figure 1B**). The predictive performance of the riskmodel was assessed using ROC analysis, and the area under curve (AUC) values for 1- and 3-yearsurvival were 0.76 and 0.66, respectively (**Figure 1C**), indicating good accuracy.

### 3.2 External validation of the DDR-gene prognostic signature

**Figure 2:**
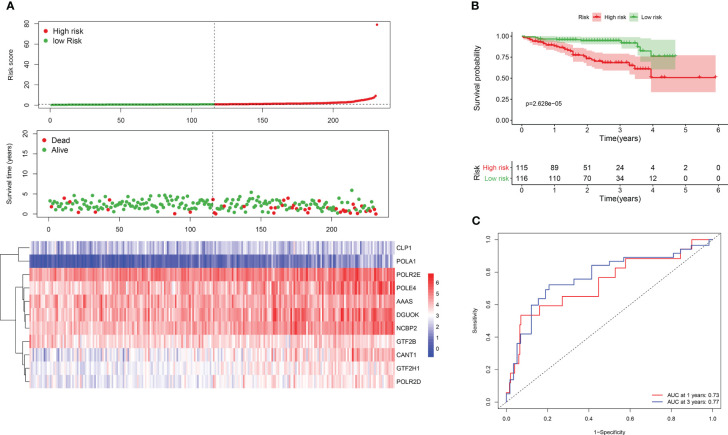
Survival analysis of DDR genes in ICGC dataset. **(A)** Risk score plot for the DDR signature. Upper panel is demonstrating the risk score distribution. Lower panel shows the level of expression of the 11 DDR genes and middle is for case distribution **(B)** KM survival curves for both groups. **(C)** ROC curve for risk gene signature.

To evaluate the reliability and robustness of the 11-gene signature, we obtained a dataset of 231 HCC samples from ICGC (https://dcc.icgc.org/). Risk scores were computed for each patient, and thecohort was divided into high- and low-risk groups. Consistent with the previous findings, themajority of surviving cases were classified into the low-risk group, while a smaller proportion ofsurviving patients were classified into the high-risk group with higher mortality rate (**Figure 2A**).KM analysis confirmed better survival outcomes for individuals in the low-risk group compared totheir high-risk counterparts (**Figure 2B**). The AUC values for 1- and 3-year survival were 0.77 and0.73, respectively, indicating a good prognostic performance of the risk model in HCC (**Figure 2C**).

### 3.3 The risk model is an independent prognostic predictor of HCC

**Figure 3:**
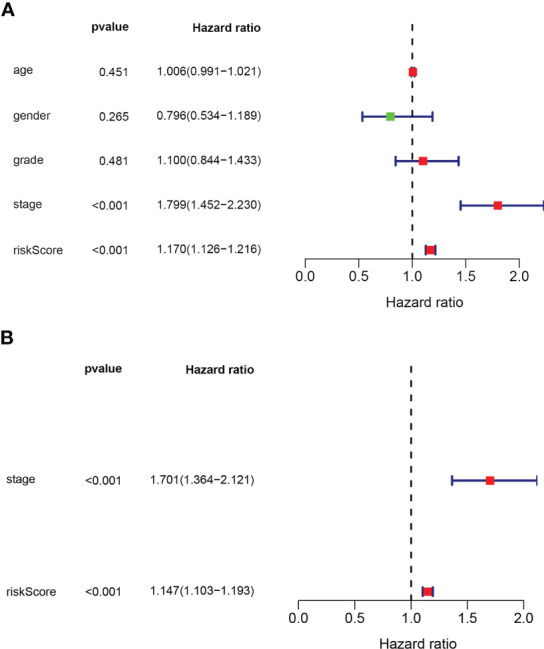
**(A)** Univariate and **(B)** multivariate cox regression analyses used to get prognostic value of the gene biosignature and clinical traits.

**Figure 4:**
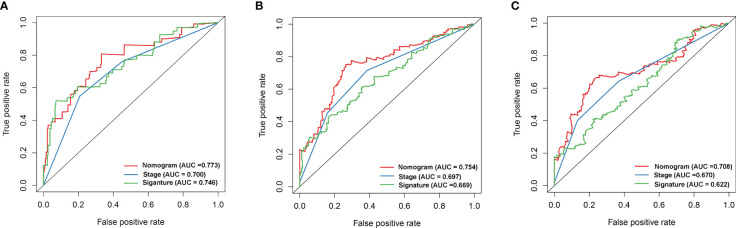
The gene signature, ROC curve analysis of the nomogram, and disease stage for 1- **(A)**, 3- **(B)**, and 5-year **(C)** survival.

**Figure 5:**
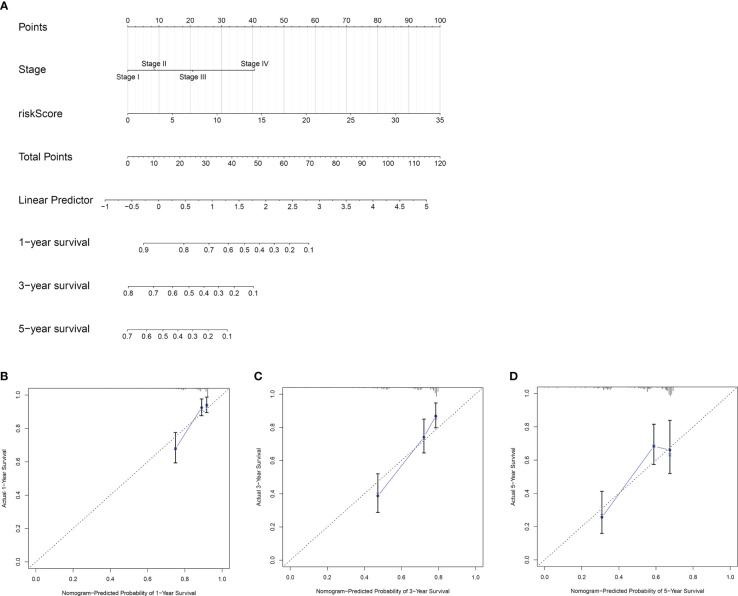
Construction and validation of a nomogram(prognostic). **(A)** A nomogram based on the risk model and tumor stage to estimate overall survival of HCC patients. The estimating of 1- **(B)**, 3- **(C)**and 5-year **(D)** survival of HCC patients using calibration curve plot of the nomogram.

Univariate and multivariate Cox regression analyses showed that the risk model and tumor stagewere independent risk factors for HCC, as reported in **Figures 3A, B**. Furthermore, ROC analysisdemonstrated that the risk model performed better than tumor stage in predicting 1-year prognosis,with AUC values of 0.746 and 0.700, respectively (**Figure 4A**). Subsequently, we constructed anomogram that integrated the risk model and tumor stage to predict overall survival (OS) at 1-, 3-,and 5-year timepoints (**Figure 5A**). The nomogram exhibited good prognostic performance, asindicated by AUC values at 1-, 3-, and 5-year timepoints (**Figures 4B, C**). The stability of thenomogram was further validated by calibration curve plots (**Figures 5B–D**). Overall, the DDR genebased risk score and tumor stage-based nomogram can robustly predict the prognosis of HCCpatients and thus, can be useful in clinical decision-making.

### 3.4 Gene set enrichment analysis

To identify enriched pathways in HCC, GSEA was conducted for both high- and low-risk groups.

**Figure 6:**
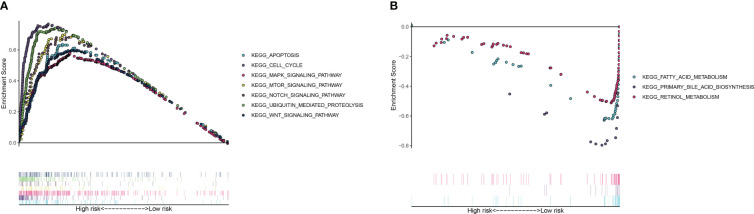
Gene set enrichment analysis results showing enriched pathways in **(A)** high-risk and **(B)** low-risk groups.

Results showed that pathways associated with apoptosis, cell cycle, and MAPK, mTOR, NOCTH, UBIQUITIN, and WNT signaling were enriched in the high-risk group, while the low-risk groupexhibited enrichment of pathways related to metabolism of fatty acid and retinol (**Figures 6A, B**).These findings suggest that favorable prognosis and low-risk scores are correlated with metabolismlinked pathways, whereas cancer-related pathways coincide with high-risk scores and poor prognosis.

### 3.5 Identification of molecular subtypes of HCC

**Figure 7:**
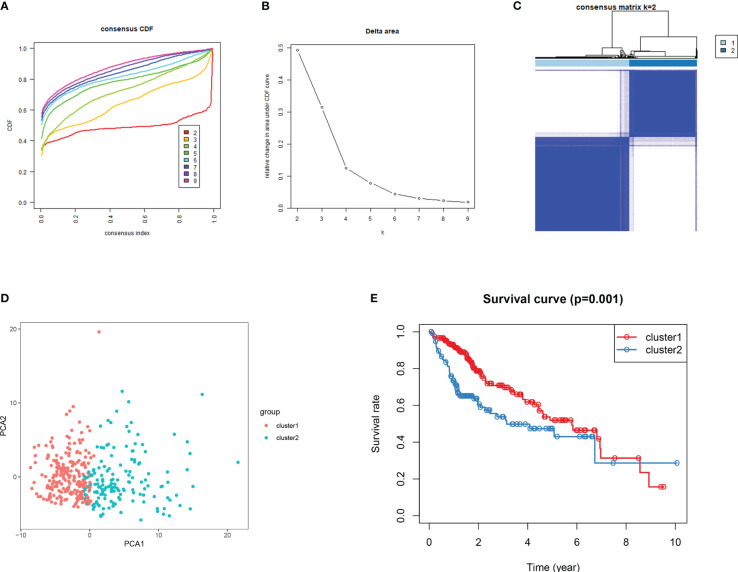
Consensus clustering for DDR genes in HCC patients from TCGA dataset. **(A)** Thecumulative distribution function (CDF) curve plot for k = 2 to k = 9 **(B)**. The change in the areaunder the CDF curve when k = 2 to k = 9. **(C)** Consensus heatmap at k =2. **(D)** Principal componentsanalysis for the DDR gene expression. **(E)** For the 2 subgroups, KM survival curve analysis.

**Figure 8:**
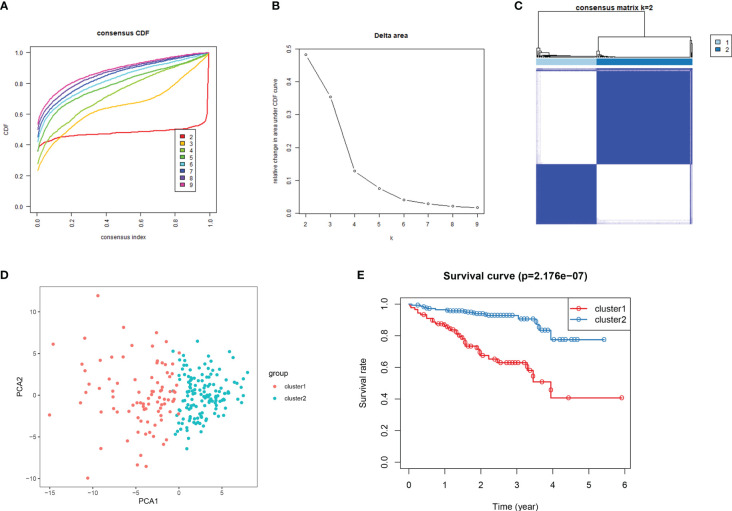
Consensus clustering for DDR genes in HCC patients from the ICGC cohort. **(A)** At k = 2 to k = 9, the cumulative distribution function (CDF) curve plot. **(B)**. The change in area under CDFcurve at two different values of k viz: k = 2 to k = 9. **(C)** At k =2, the consensus heatmap **(D)** Principal components analysis for the expression of DDR gene **(E)** KM survival curve analysis forthe 2 subgroups.

**Figure 9:**
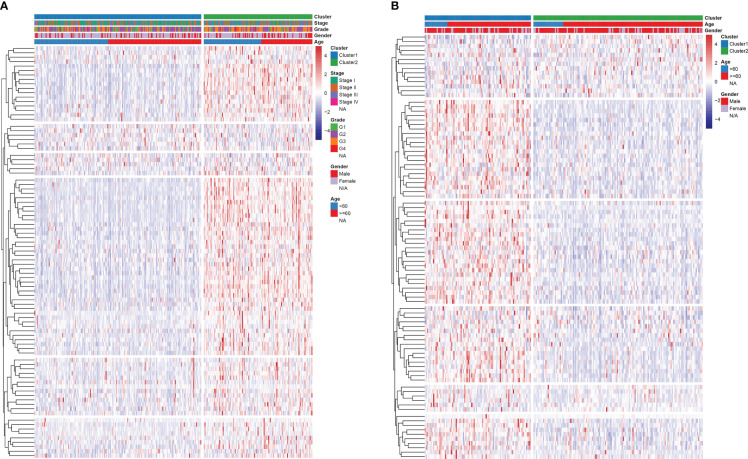
Heatmap analysis of the relationship between subgroup and clinical traits in **(A)** TCGA and **(B)** ICGC cohorts.

Consensus clustering algorithm based on the DDR genes was employed to identify the molecularsubtypes of HCC. The optimal cluster was determined to be K=2 using the cumulative distributionfunction curve and the consensus heatmap (**Figures 7A–C**). PCA further differentiated patients intotwo distinct subgroups (**Figure 7D**), with subgroup 1 exhibiting better overall survival compared tosubgroup 2 (**Figure 7E**). To validate the robustness of the classification, we also evaluated thesubgroups in the ICGC dataset (**Figure 8**). Further analysis of the correlation and clinicalcharacteristics in both TCGA and ICGC datasets for the two subgroups showed that the group withbetter survival outcomes included more early-stage cases (**Figures 9A, B**).

### 3.6 Correlation of the immune infiltration with HCC subclasses

**Figure 10:**
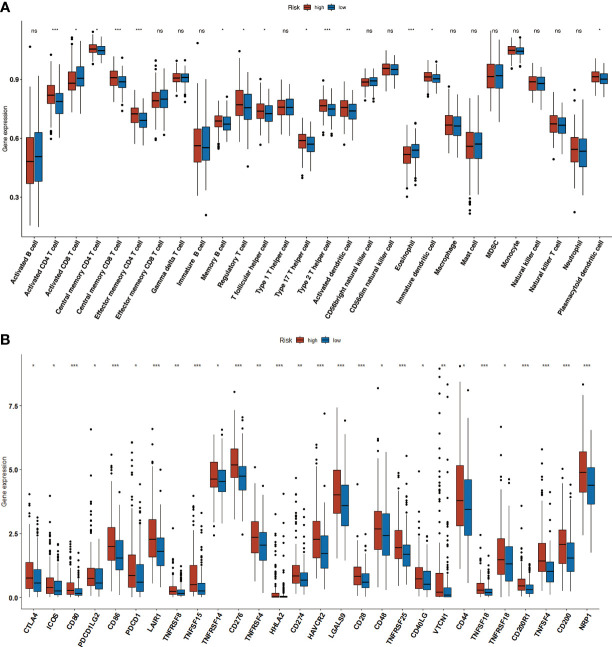
Immune infiltration. **(A)** The increased number of tumor infiltrating immune cells in high-and low-risk groups. **(B)** Boxplot showing the immune-checkpoint genes expression in low- andhigh-risk groups. * p<0.05, ** p <0.01, *** p<0.001.

The ssGSEA algorithm was used to analyze the infiltration of 24 immune cells in both the high- and low-risk groups. The high-risk group showed high levels of infiltration of activated CD4+ T cells,CD4+ T (central memory) CD8+ T cells (central memory), CD4+ T cells (effector memory), B cells(memory), regulatory T cells, T follicular helper cells, Th17 cells, Th2 cells, activated CD8+ T cells,immature dendritic cells (DCs), and plasmacytoid DCs, whereas the low-risk group had greaterinfiltration of activated CD8+ T cells and eosinophils (**Figure 10A**). Additionally, the high-risk group exhibited comparatively higher expression of all inhibitory immune receptors compared to the low-risk group (**Figure 10B**). These findings suggest that the anti-tumor properties of high T cell infiltration were offset by a strong immunosuppressive tumor microenvironment due to the overexpression of immune checkpoint proteins (18).

### 3.7 Cell assays

**Figure 11:**
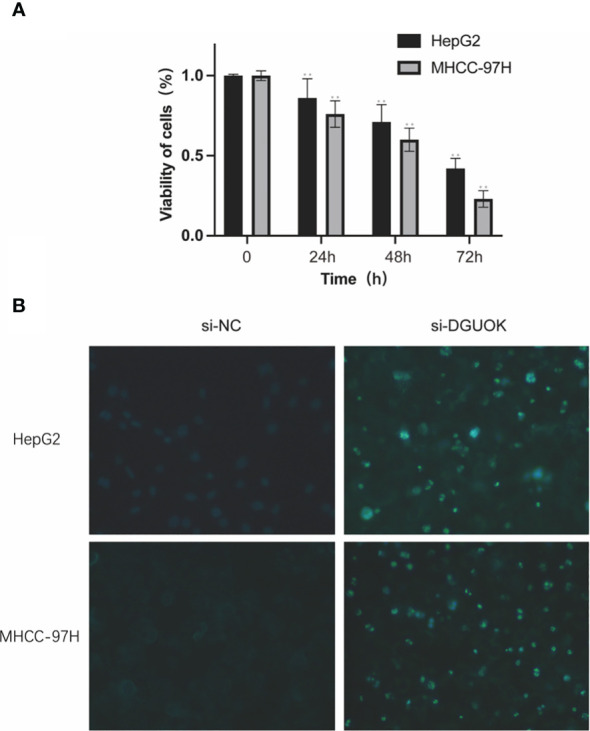
*In vitro* validation on DGUOK. HepG2 and MHCC-97H cells were treated with siDGUOK for indicated time. **(A)**: CCK8 was performed to detect the cell viability. **(B)**: Hoechst 33258 fluorescent dye staining was used to show nuclear morphological changes and to assess apoptosis. Data in **(A)** are presented as means ± SD. **p< 0.01. magnification: 200×.

*In vitro* validation on DGUOK. HepG2 and MHCC-97H cells were treated with CCK8 and were performed to detect the cell viability. Hoechst 33258 fluorescent dye staining was used to show nuclear morphological changes and to assess apoptosis. Data in A are presented as means ± SD. **, p< 0.01. magnification: 200×. The results showed the proliferation ability is higher in the si-NC group compared by the si-DGUOK group (**Figure 11**).

### 3.8 MiRNA-mRNA Network

**Figure 12:**
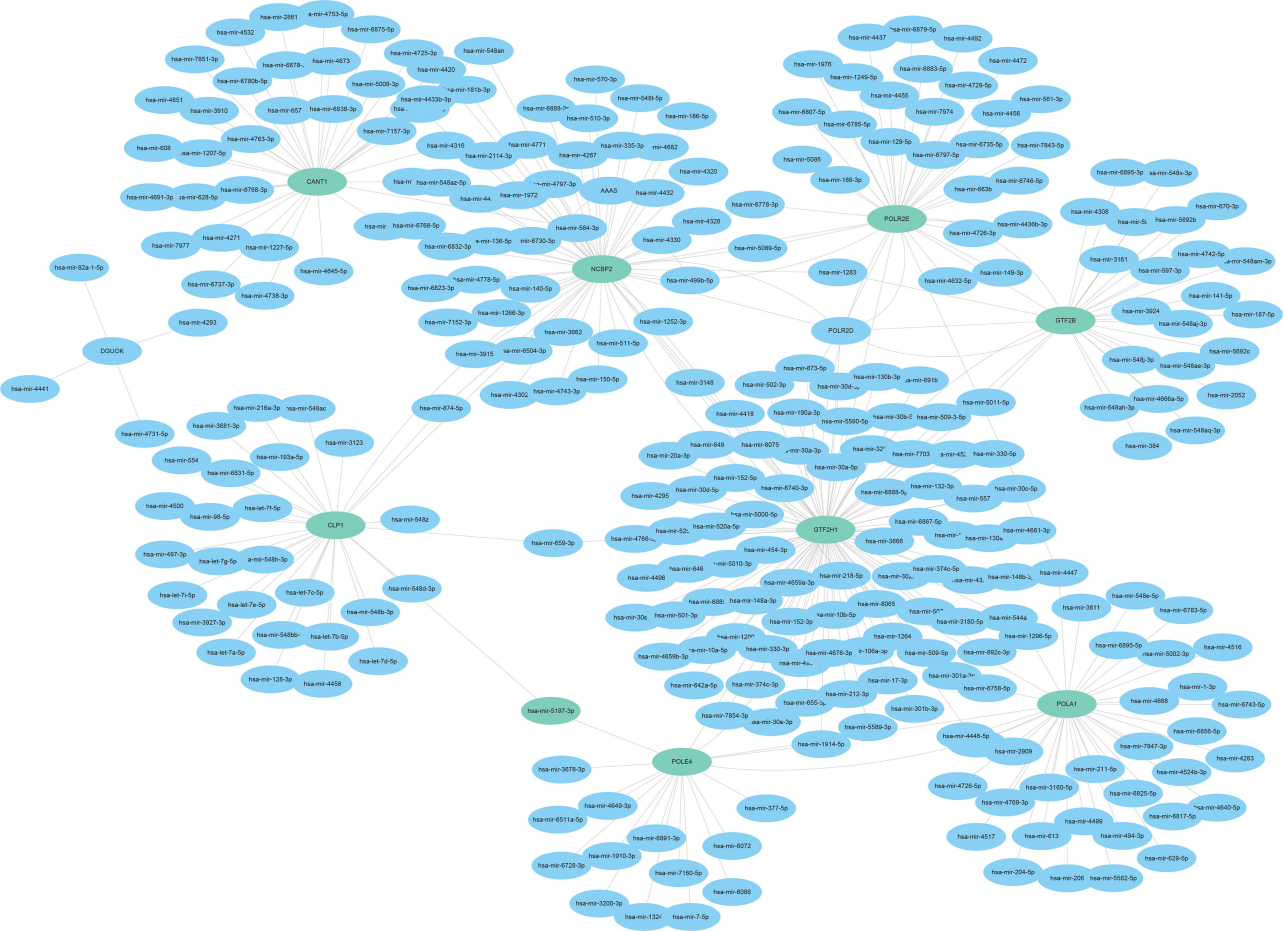
DNA Repair Gene miRNA-mRNA Network.

Based on the targetscan database data, we performed a relevant microRNA analysis of DNA repair genes in Siganture using the Cytoscape software. and conducted DNA repair related miRNA-mRNA Network (**Figure 12**).

## 4 Discussion

HCC is a highly heterogeneous cancer with multiple risk factors, including alcohol consumption, hepatitis B/C infection, and obesity (19). The initiation of HCC is associated with DNA damage and chromosomal abnormalities, which triggers a DNA damage response (DDR) in affected cells. DNA lesions can be repaired through various mechanisms, including homologous recombination, mismatch repair, and double-strand break repair. Dysfunctional DDR pathways can result in genomic instability, mutations, and eventually lead to HCC development and progression (20). Many DNA repair proteins, such as sphingolipid signaling, TP53, hOGG1, XRCC1, PARP-1, MRE11-Rad50NBS1 (MRN) complex, and ataxia-telangiectasia mutant (ATM) kinase, are frequently mutated in HCC (21). Furthermore, ionizing radiation (IR)-induced DDR pathways can create an immunosuppressive tumor microenvironment, thereby weakening the anti-tumor effect of radioimmunotherapy. DDR inhibitors can reverse the immunosuppressive state of HCC and inhibit tumor progression, providing a potential therapeutic strategy (22).

There is a substantial body of evidence suggesting that DNA damage response (DDR) genes play a crucial role in the development of cancer (23). DDR genes are often expressed abnormally in mucosal or tumor tissues and are closely associated with patient prognosis (24, 25). However, the ability of individual genes to serve as prognostic markers is limited (26, 27), and multi-gene signatures may be better suited for predicting the prognosis of hepatocellular carcinoma (HCC). Despite this, no study has yet investigated the prognostic value of DDR genes in HCC. To address this gap, we developed an 11-gene signature based on DDR gene expression data and clinical data obtained from the ICGC and TCGA databases. The risk score generated by the 11-gene signature enabled the classification of patients into low- and high-risk groups, with the latter group exhibiting poorer survival outcomes. The risk model demonstrated good predictive performance in both TCGA and ICGC datasets. Additionally, the risk model was found to be an independent prognostic factor for HCC. A nomogram constructed using the risk score and tumor stage allowed the clear differentiation of two prognostic groups, which may be helpful in guiding preoperative management of HCC patients. The DDR gene signature identified in this study was found to be linked with several cancer related pathways including cell cycle, WNT signaling, mTOR signaling and apoptosis in the high risk group, which may be indicative of the potential mechanisms underlying HCC progression. On the other hand, the low-risk group was enriched in metabolism-related pathways. Most of the genes in the DDR-based risk signature have been implicated in tumorigenesis. For instance, CANT1 is known to regulate pyrimidine metabolism in melanoma cells and is associated with tumor progression (28). High expression of CANT1 in prostate cancer cells has been associated with better prognosis, while its silencing significantly suppressed cell proliferation and DNA synthesis (29). CLP1, on the other hand, plays an important role in motor neuron function (30). Mitochondrial deoxyguanosine kinase (DGUOK) is an enzyme that controls the rate of deoxy nucleoside salvage pathway in the mitochondria. Overexpression of DGUOK has been associated with worse prognosis in lung cancer, and its depletion suppressed lung adenocarcinoma growth, CSC self-renewal and metastasis (31). GTF2B has been identified as a prognostic marker for colorectal cancer and neuroblastoma, while GTF2H1 is a p62 subunit of complex transcription factor IIH (TFIIH) that regulates nucleotide excision repair and transcription (32, 33). Certain polymorphisms/haplotypes of GTF2H1 have been associated with increased susceptibility to lung cancer (34). Additionally, the budding yeast orthologs of POLE4 have been shown to enhance Polϵ processivity *in vitro*, but have the opposite effect *in vivo*, leading to accelerated tumorigenesis (35).

Moreover, the POLR2E rs3787016 polymorphism may enhance the risk of developing the prostatecancer, liver cancer esophageal cancer, papillary thyroid carcinoma, and breast cancer (36–38). However, the role of AAAS, NCBP2, POLA1 and POLR2D in HCC is unknown, and will have to beexperimentally verified. Immunotherapy has achieved encouraging results in various malignancies (39), including HCC. For instance, the “T+A” scheme is increasingly becoming the first-line optionfor advanced HCC (40). Despite achieving good outcomes in multiple cancers, a significantpercentage of the patients do not benefit from immunotherapy. Hence it is necessary to recognize thebiomarkers that can reveal the outcomes of immunotherapy, and screen for patients that can respondto immunotherapeutic regimens. Galon et al. (41) had proposed the concept of “cold” and “hot” tumors to evaluate their sensitivity to immunotherapies. In this study, we detected increased infiltration of immunosuppressive cells and overexpression of receptors responsible for immune checkpoint in the high-risk group, which indicates that the high-risk group patients are likely unresponsive to immunotherapy.

In summary, we identified biomarkers of HCC based on computational biology in oncologymethods (42, 43), and constructed prognostic models using machine learning methods (44–46). Wehave established an 11-DDR gene signature that can accurately forecast the prognosis ofhepatocellular carcinoma (HCC). The utilization of this prognostic signature not only advances our comprehension of the underlying molecular mechanisms that contribute to HCC progression but also provides a practical guide for clinical decision-making.

## Data availability statement

The original contributions presented in the study are included in the article/supplementary material. Further inquiries can be directed to the corresponding author.

## Ethics statement

The studies involving human participants were reviewed and approved by Intensive Care Medicine, Liaoyou Baoshihua Hospital. The patients/participants provided their written informed consent to participate in this study.

## Author contributions

YB contributed to data analysis, methodology, figures construction, and article writing. YWcontributed to investigation and validation. JH, YM, HL and ML contributed to methodology and validation. All authors contributed to the article and approved the submitted version.
